# Estimation of cognitive impairment in chronic pain patients and characteristics of estimated mild cognitive impairment

**DOI:** 10.3389/fneur.2024.1344190

**Published:** 2024-03-05

**Authors:** Masamichi Moriya, Lizhen Hu, Kaoru Sakatani, Masaki Kitahara

**Affiliations:** ^1^Department of Autonomic Neuroscience, Tokyo Metropolitan Institute of Gerontology, Tokyo, Japan; ^2^Department of Human and Engineered Environmental Studies Graduate School of Frontier Sciences, The University of Tokyo, Kashiwa, Japan; ^3^Department of Anesthesiology, Yokohama City University Medical Center, Yokohama, Kanagawa, Japan

**Keywords:** chronic pain, multidisciplinary pain clinic, mini mental state examination, deep neural network, physical activity

## Abstract

**Background:**

Patients with chronic pain suffer from psychological effects such as anxiety due to the pain itself. Pain can not only impair activities of daily living (ADL) and quality of life (QOL), but also impair cognitive function. Therefore, in this study, we aimed to estimate the cognitive function of chronic pain patients using a deep neural network (DNN) model that has already been implemented in society. We investigated the characteristics of patients presumed to have mild cognitive impairment (MCI) and, at the same time, verified the relationship with the questionnaire commonly used in chronic pain research, which is administered by 43 university affiliated hospitals and medical institutions participating in the chronic pain research group of the Ministry of Health, Labor and Welfare in Japan (assessment batteries).

**Method:**

The study included 114 outpatients from a multidisciplinary pain clinic, and we estimated their Mini-Mental State Examination (MMSE) scores based on age and basic blood test data (23 items). Furthermore, we classified the estimated MMSE scores of chronic pain patients into two groups based on a cutoff score of 27, which indicates MCI, and compared the blood data and assessment batteries. Additionally, we used a control group of 252 healthy adults aged 45 years or older who visited a dementia prevention outpatient clinic for comparison with the MMSE scores of chronic pain patients.

**Result:**

The MMSE scores in chronic pain patients were below the cutoff for MCI. When classified into two groups based on the estimated MMSE score of 27 points, WBC, RBC, Hb, Hct, PLT, UA, BUN, creatinine, Triglyceride, and γ-GT were significantly higher in the blood data. In the MCI group, PDAS values were significantly lower. Furthermore, only in the non-MCI group, a significant correlation was found between the estimated MMSE value and BPI, PDAS, and Locomo. The estimated MMSE scores were significantly lower in chronic pain patients than in healthy adults (*p* = 0.04).

**Conclusion:**

Patients with chronic pain may exhibit cognitive impairment due to systemic metabolic disturbances. This suggests that chronic pain affects activities of daily living, resulting in systemic metabolic disorders.

## 1 Introduction

The economic burden in our nation due to chronic pain is estimated at approximately $1.3 million (2 trillion yen) and has recently emerged as an urgent societal concern (1, 2). Treatment resistance in chronic pain is attributed to alterations in the central (brain) regions, with an overexcitation of the amygdala and inactivation of the prefrontal cortex being implicated. Among these, the “amygdala-driven inactivation,” signifying the absence of interaction between the amygdala and prefrontal cortex, has been reported to impair not only physical functions but also cognitive functions in response to pain (3). Epidemiological analyzes show that at least 50% of people living with pain have cognitive problems (4). Other reports suggest the possibility of structural and functional brain changes induced by pain (5). Consequently, living with chronic pain may lead to an accumulation of brain pathologies associated with dementia, becoming a burden, and compromising the brain’s resilience (6). Growing evidence suggests patients with chronic pain or Alzheimer’s disease exhibit abnormalities of the noradrenergic (NE) system in the locus coeruleus (LC) (7), activation of microglia in brain regions such as the frontal cortex, and increased central nervous inflammation in these regions (8). Chronic pain induces pathological activation of the LC-NE system, resulting in increased NE release in brain regions such as the prefrontal cortex and hippocampus. This may be one of the mechanisms of chronic pain-induced proinflammatory activation of microglia. Proinflammatory activation may exacerbate AD pathogenesis through decreased Aβ phagocytosis, increased tau dissemination, loss of synaptic function, and cytokine-induced neuronal death in these brain regions (9). However, research also exists that indicates no direct relationship between pain and cognitive dysfunction (10), and thus, a consensus on the interrelationship between chronic pain and cognitive impairment remains elusive.

Our research group has developed and applied a deep neural network (DNN) model that can predict cognitive impairment by analyzing the intricate non-linear relationship between systemic metabolic abnormalities and cognitive function using basic blood test data (11). Metabolic disorders, such as malnutrition, anemia, lipid metabolism, purine metabolism, and kidney function impairment, which may potentially influence cognitive function, can be detected in routine health assessments through basic blood tests. Recent developments have reported various extensions of this model, including the possibility of model reconstruction with the addition of Near-infrared spectroscopy (NIRS) data (12) and the estimation of brain atrophy using the same model (13).

In this study, our objective is to validate the hypothesis that patients experiencing a decline in their daily activities due to pain exhibit a significant reduction in their quality of life (QOL) and, because of disrupted lifestyle habits, induce systemic metabolic changes that impair cognitive function. We used an already socially implemented DNN model to estimate the cognitive function of chronic pain patients. In addition to exploring the potential for cognitive impairment resulting from structural and functional changes in the brain, we simultaneously examined the possibility of cognitive impairment due to systemic metabolic changes. Furthermore, we conducted additional investigations to identify the characteristics of patients who visited the Pain Clinic Department and were estimated to have mild cognitive impairment (MCI). We also examined the relationship with the common questionnaire (assessment battery) used in Japan by the Ministry of Health, Labor and Welfare’s Chronic Pain Research Group, which is shared among 43 universities and medical institutions (14, 15) (Table 1).

**Table 1:** Estimated MMSE for pain patients.

Blood test items		Assessment battery
Complete blood count	General biochemical examination	
WBC count (10^3/μL)	Total protein (g/dL)	BPI (worst)
RBC count (10^6/ μL)	Albumin/Globulin ratio	BPI (current)
Hemoglobin (g/dL)	Albumin (mg/dL)	PDAS
Hematocrit (%)	Uric Acid	HADS-Anxiety
Platelet count (10^3/μL)	blood urea nitrogen (mg/dL)	HADS-Depression
MCV	Creatinine (mg/dL)	PCS
MCH	Na	EQ5D
MCHC	K	PSEQ
	Cl	AIS
	Total Cholesterol(mg/dL)	Locomo
	Triglyceride (mg/dL)	
	AST	
	ALT	
	γーGT	
	Glucose	

MCV, mean corpuscular volume; MCH, mean corpuscular hemoglobin; MCHC, mean corpuscular hemoglobin concentration; AST, Aspartate Aminotransferase; ALT, Alanine Aminotransferase; γ-GTP, Gamma-Glutamyl Transferase.

## 2 Methods

### 2.1 Subjects

The subjects of this study consisted of 114 cases (48 males, 66 females) who received physiotherapy-guided exercise therapy at our Pain Clinic Department. Inclusion criteria encompassed patients with chronic pain in various parts of the body, including myofascial low back pain. Exclusion criteria were individuals who had not undergone blood tests and those with blank responses on the questionnaire. For comparative purposes with chronic pain patients, data from 252 cases (122 males, 130 females) who visited the Dementia Prevention Outpatient Clinic at Tokyo Clinic (Chiyoda-ku, Tokyo) were used as the control group. The control group comprised healthy adults aged 45 or above, capable of outpatient visits and independent in their daily activities. This study was approved by the Ethics Committee for Research in the Life Sciences and Medicine involving individuals at Yokohama City University (Approval Number: F230400058). This study is an observational study conducted at a single institution without any invasiveness or intervention, using only existing information based on anonymous data from medical records. Therefore, the requirement for informed consent was waived for all subjects, but information about the research was made public and research subjects were guaranteed the opportunity to refuse.

### 2.2 Adaptation of DNN model by blood test

**Figure 1:**
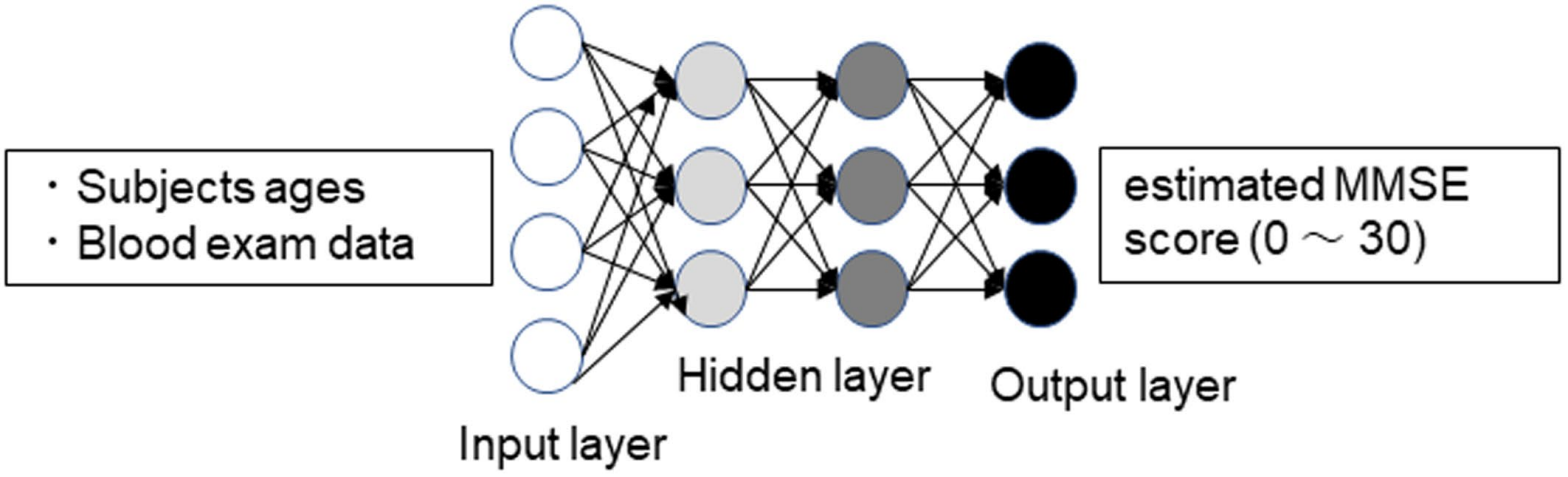
Schematic drawing of the DNN model for predicting MMSE scores. Input vectors include subject age and blood examination data. The output vector is a regression to estimate the MMSE score (11).

All subjects underwent basic blood tests, including complete blood cell counts and a basic metabolic panel. The test was performed for medical purposes, not research. The measured parameters included WBC (White Blood Cell count), RBC (Red Blood Cell count), Hb (Hemoglobin), Hct (Hematocrit), PLT (Platelet count), TP (Total protein), A/G (Albumin/Globulin ratio), Albumin, UA (Uric Acid), BUN (blood urea nitrogen), Cr (Creatinine), Na, K, Cl, T-chol (Total Cholesterol), TG (Triglyceride), AST (Aspartate Aminotransferase), ALT (Alanine Aminotransferase), γ-GTP (Gamma-Glutamyl Transferase), and GLU (Glucose). Chronic pain patients completed blood tests at their initial consultation. Patients who had not undergone blood tests had them conducted during their first visit to the Pain Clinic Department. The blood test data were input into a DNN model that had already been socially implemented to estimate the Mini-Mental State Examination (MMSE) score (11) (Figure 1). To implement the DNN, we used the H2O open-source machine learning library (16, 17). H2O allows the configuration of multilayer feedforward neural networks. Input to the DNN in this study consisted of 25 variables, including subject age plus 23 blood test items in the input layer. The DNN has two hidden layers with 400 neurons each. The weighted combination α = $\sum_{i=1}^{n}w_{i}x_{i}+b$ aggregates the input signals χ *_i_* in each layer to activate an output signal $f\left(\alpha \right)$ to the connected neuron in the next layer. The function f represents the non-linear activation function used throughout the network, and the bias *b* accounts for the neuron’s activation threshold. The output signal $f\left(\alpha \right)$ in each layer is thus determined by the weighted combination with the input signals χ *_i_* from the higher layers of the DNN. In the output layer, a loss function *L* (*W, B |j*) is measured using the mean square error between the estimated value and the actual MMSE score. The learning process updates the weights *W* and biases *B* until the loss function *L* (*W, B |j*) is minimized. We use W to denote the collection {*W i*}_1: N-1’_where *Wi* denotes the weight matrix connecting layers *i* and *i* + 1 for a network of *N* layers. Similarly, *B* denotes the collection {*bi*} _1: N -1′_ where *bi* denotes the column vector of biases for layer *i* + 1. The algorithm was validated by Leave-one-out Cross Validation.

### 2.3 Assessment battery for 43 universities and medical institutions participating in the chronic pain research group of the ministry of health, labor, and welfare (assessment battery)

In this study, a common questionnaire used nationwide in Japan was administered to all patients during their initial consultation. These questionnaires are collectively evaluated and utilized in 43 facilities of universities and medical institutions in Japan specializing in pain management. The Brief Pain Inventory (BPI) assesses the severity of pain on a 10-point scale (18). In this study, two items were used: the most intense pain experienced in the last 24 h and the current pain level. The Pain Disability Assessment Scale (PDAS) evaluates the extent to which chronic pain patients’ daily life, particularly their physical activity and mobility, is impaired (19). The Hospital Anxiety and Depression Scale (HADS) assesses the levels of anxiety and depression in the subjects (20). The Pain Catastrophizing Scale (PCS) measures the degree of catastrophic thinking and consists of three factors with 13 items, evaluating the extent to which they apply to the subject’s typical pain experience on a 5-point scale (21). The EuroQol-5D (EQ-5D) is an assessment that involves patients reporting their own health status, and utility values are obtained using a conversion table (22). The Pain Self-Efficacy Questionnaire (PSEQ) evaluates self-efficacy regarding pain with 10 items, each rated on a 7-point scale (23). The Athens Insomnia Scale (AIS) evaluates the severity of insomnia with eight items, each rated on a 4-point scale (24). The 25-question Geriatric Locomotive Function Scale (Locomo) is composed of 25 questions used as a diagnostic tool for locomotive syndrome, with scores ranging from 0 to 100 on a 5-point scale indicating the most severe condition (25).

### 2.4 Data analysis and statistical analysis

Initially, the estimated MMSE values of chronic pain patients were calculated, classified the estimated MMSE scores of chronic pain patients into two groups based on a cutoff score of 27, which indicates MCI. Blood test data were compared between these groups. To investigate the relationship between estimated MMSE values and blood test data, multiple regression analyses were conducted. Bubble plots were designed to display clinically significant information in three dimensions (estimated MMSE score, hemoglobin, triglyceride). To examine the relationship between questionnaire results and cognitive function, subjects were classified into two groups based on the MMSE cutoff value and subjected to comparative analysis, along with correlation analysis. Lastly, estimated MMSE values were compared between chronic pain patients and the control group.

## 3 Results

### 3.1 Estimated MMSE scores of chronic pain patients

Using the DNN model, we calculated estimated MMSE scores based on the initial blood data of all chronic pain patients. Analysis of data from all 114 patients revealed a median MMSE score of 26.8, falling below the cutoff value for Mild Cognitive Impairment (MCI). Upon classification using the cutoff, the MCI group (<27 points) consisted of 62 cases (54.4%), while the non-MCI group (≥27 points) comprised 52 cases (45.6%), indicating that over half of the chronic pain patients were estimated to have MCI. Analysis of the age distribution showed that the MCI group was significantly older, with the non-MCI group mainly consisting of individuals in their 50s and below (Table 2). In other words, half of chronic pain patients have the possibility of MCI, suggesting that the possibility of MCI is particularly high in the elderly.

**Table 2:** Estimated MMSE for pain patients.

		All	MCI group	non-MCI group	*p*
*n* (%)		114 (100)	62 (54.4)	52 (45.6)	
Estimated MMSE, Median (IQR)		26.8 (25.6–27.8)	25.7 (24.3–26.5)	27.9 (27.5–28.2)	
Age, *n* (%)	<50	28	8	20	0.0001
	50-	41	18	23	
	60-	24	17	7	
	70-	15	13	2	
	80-	6	6	0	

### 3.2 Characteristics of blood data in chronic pain patients and multiple regression analysis

**Figure 2:**
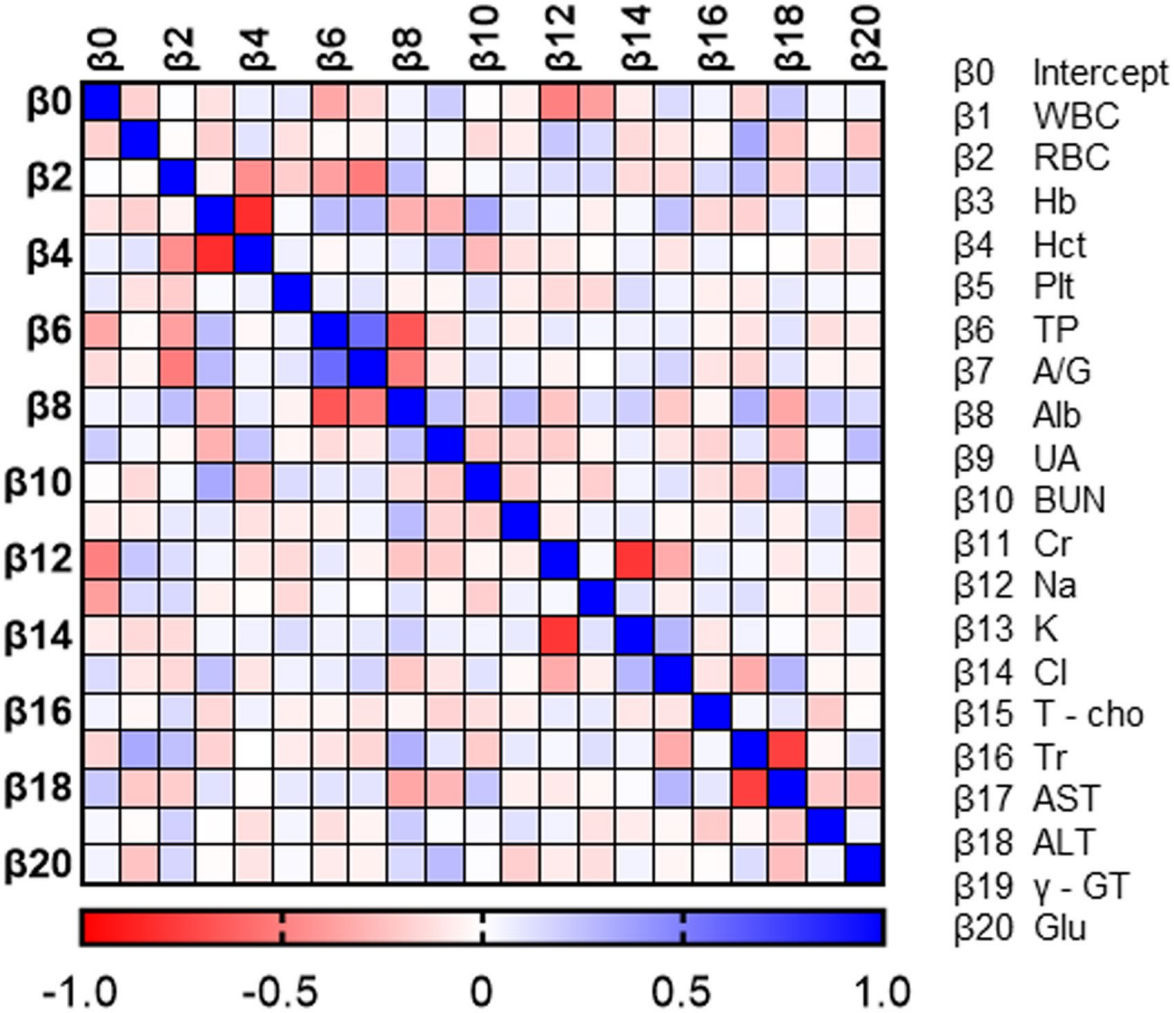
Correlation heat map of blood data. Each value in the normalized covariance matrix ranges from −1.0 to 1.0. A value equal to −1.0 or 1.0 means the two parameters are redundant.

**Figure 3:**
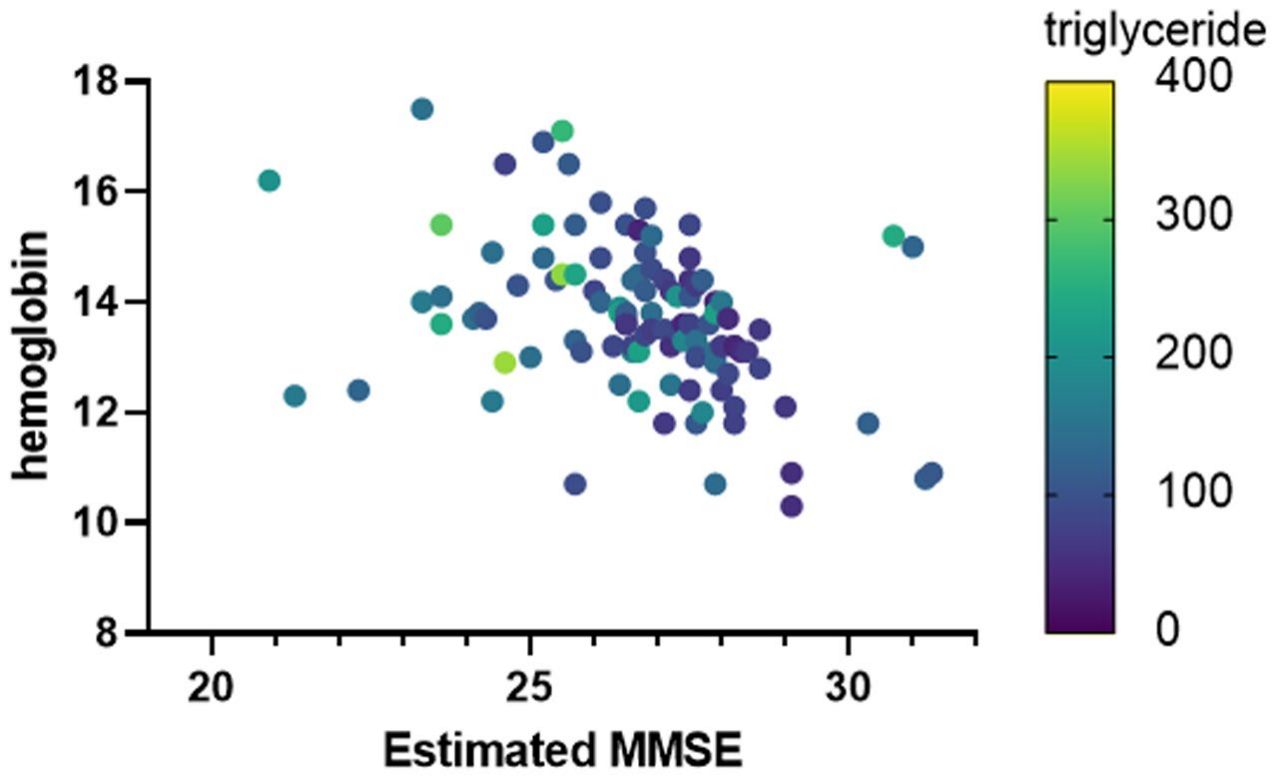
Clinically important factors associated with estimated MMSE scores. The horizontal axis is the estimated MMSE score. The vertical axis shows the hemoglobin value, and the color of the bubble shows the Triglyceride value.

To verify the characteristics of patients estimated to have MCI, we compared the blood data underlying the DNN model between the MCI group and the non-MCI group. In the MCI group, several blood parameters were significantly elevated, including WBC (*p* = 0.0124), RBC (*p* = 0.0124), Hb (*p* = 0.0001), Hct (*p* = 0.0001), PLT (*p* = 0.0206), UA (*p* = 0.0028), BUN (*p* = 0.004), Creatinine (*p* = 0.0003), Triglyceride (*p* = 0.0001), and γ-GT (*p* = 0.0015) (Table 3). Furthermore, to investigate the relationship with the estimated MMSE score, a multiple regression analysis was conducted, encompassing all blood test parameters. The results demonstrated an ability to explain 70.4% of the estimated MMSE score (*R*^2^ = 0.704). Significant influencing factors extracted from this analysis included Platelet (β = 0.01945, *p* = 0.0004), BUN (β = −0.1614, *p* = 0.0076), Na (β = 0.5633, *p* = 0.0003), and Triglyceride (β = −0.00659, *p* = 0.0008) (Table 4). To visualize clinically significant information from the blood data, a bubble plot was produced using three dimensions (estimated MMSE score, hemoglobin, triglyceride) (Figure 2). Hemoglobin values were mostly above 12, with some patients showing a tendency toward anemia at around 12. For Triglyceride, patients with values above the MCI cutoff of 27 were predominantly represented in dark blue, while those with values below 26 displayed shades ranging from green to light green. Finally, we investigated the extent to which each item related to the blood data of chronic pain patients. Covariance analysis showed strong correlations between Hb and Hct (−0.826), Na and Cl (−0.790), and AST and ALT (−0.749) (Figure 3).

**Table 3:** Blood data characteristics of the patients based on the estimated MMSE.

Pain patients	Estimated MMSE			
	*n* = 62	*n* = 52		
	MCI group	non-MCI group	*p*	
**WBC count (10^3/μL)**	**5,725**	**5,210**	**0.0124**	*
**RBC count (10^6/ μL)**	**464.5**	**438.0**	**0.0124**	*
**Hemoglobin (g/dL)**	**14.1**	**13.2**	**0.0001**	****
**Hematocrit (%)**	**42.9**	**40.0**	**0.0001**	****
**Platelet count (10^3/μL)**	**23.25**	**24.75**	**0.0206**	*
Total protein (g/dL)	7.2	7.2	0.294	
A/G	1.595	1.655	0.139	
Albumin (mg/dL)	4.4	4.5	0.4812	
**UA**	**5.2**	**4.4**	**0.0028**	**
**BUN (mg/dL)**	**15.5**	**13**	**0.004**	**
**Creatinine (mg/dL)**	**0.79**	**0.665**	**0.0003**	***
Na	142	142	0.815	
K	4.2	4.2	0.4729	
Cl	104	105	0.2251	
Cholesterol (mg/dL)	198	215	0.655	
**Triglyceride (mg/dL)**	**132**	**83.5**	**0.0001**	****
AST	24	20.5	0.123	
ALT	19	20	0.7198	
**γーGT**	**27**	**17**	**0.0015**	**
GLU	100	97	0.0894	

Blood test data were compared between these groups (Mann–Whitney test). Important variables (*p* < 0.05) are highlighted in bold.

**Table 4:** Multiple regression analysis of variables with regard to cognitive function.

	*β*	Std Error	95% CI	*t*	*p*	
Platelet count (10^3/μL)	0.01945	0.005266	0.00896 to 0.02994	3.694	0.0004	***
BUN (mg/dL)	−0.1614	0.05878	−0.2785 to −0.0442	2.745	0.0076	**
Na	0.5633	0.1471	0.2701 to 0.08565	3.829	0.0003	***
Triglyceride (mg/dL)	−0.006591	0.00188	−0.01034 to −0.00284	3.503	0.0008	***

### 3.3 Comparison and correlation of initial questionnaire results with estimated MMSE scores

**Figure 4:**
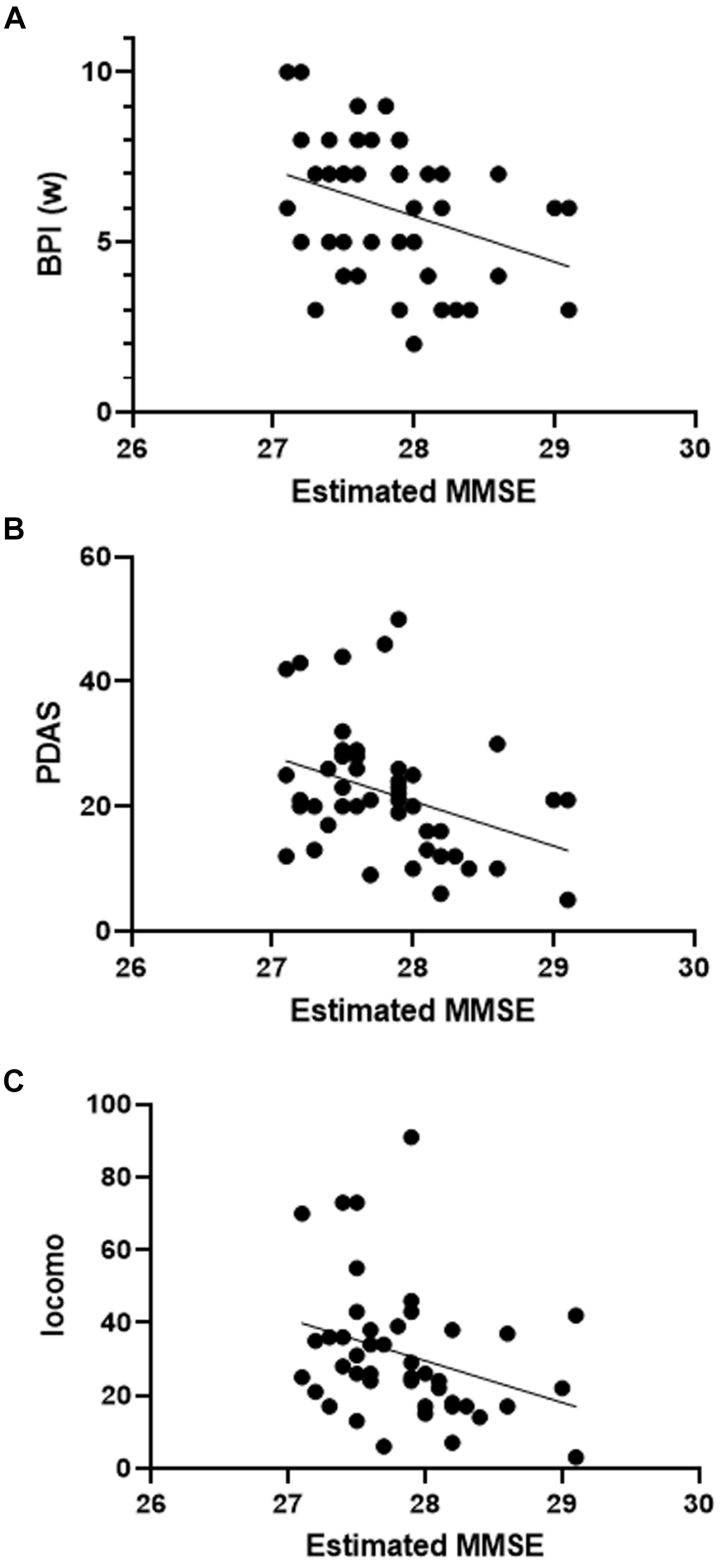
Scatter plot of estimated MMSE and assessment battery. Correlation between estimated MMSE score and assessment batteries (simple linear regression). **(A)** BPI (w): *F* = 5.748, *p* = 0.0208, *R*^2^ = 0.12. **(B)** PDAS: *F* = 6.475, *p* = 0.0144, *R*^2^ = 0.13. **(C)** Locomo: *F* = 4.679, *p* = 0.0361, *R*^2^ = 0.10.

For the purpose of examining the relationship between questionnaire results and cognitive function, data from the MCI group and non-MCI group were compared, and correlation analysis was conducted. In the MCI group, PDAS values were significantly lower (Table 5). There were no significant differences in other assessment battery. Additionally, only in the non-MCI group, a significant correlation was observed between the estimated MMSE score and BPI (worst), PDAS, and Locomo (Figures 4A–C). BPI had no correlation with “current” but had a correlation with “worst.” This suggests a potential relationship between the worst severity of pain, its impact on daily life, the degree of impaired mobility due to physical function decline, and cognitive function.

**Table 5:** Assessment battery characteristics of the patients based on the estimated MMSE.

Pain patients	MCI group	Correlation to MMSE score	non-MCI group	Correlation to MSE score	Difference (MCI vs. non-MCI)	
	*n* = 57		*n* = 46		*p*	
**BPI (worst)**	6.0 (4.0–8.0)	n.s.	6.0 (4.0–7.0)	*0.12	0.876	
BPI (current)	3.5 (2.0–6.0)	n.s.	5.0 (3.0–6.0)	n.s.	0.0974	
**PDAS**	16.5 (8.8–23.5)	n.s.	20.5 (13.8–26.0)	*0.13	0.0402	*
HADS-anxiety	7 (4.0–10.3)	n.s.	8.0 (6.0–11.8)	n.s.	0.1617	
HADS-depression	7 (4.8–11.0)	n.s.	8.0 (5.3–11.0)	n.s.	0.3445	
PCS	31.5 (23.8–40.3)	n.s.	33.5 (25.3–43.0)	n.s.	0.2017	
EQ5D	0.66 (0.53–0.77)	n.s.	0.65 (0.50–0.75)	n.s.	0.428	
PSEQ	26.5 (20.8–36.0)	n.s.	26 (14.3–37.0)	n.s.	0.4147	
AIS	7 (3.8–11.3)	n.s.	7 (5.0–10.8)	n.s.	0.8744	
**Locomo**	22.5 (17.5–34.8)	n.s.	26 (17.0–38.0)	*0.10	0.2089	

Subjects were classified into two groups based on the MMSE cutoff value and subjected to comparative analysis (Mann–Whitney test), along with correlation analysis (simple linear regression). ^*^p < 0.05. Important variables (*p* < 0.05) are highlighted in bold.

### 3.4 Comparison between chronic pain patients and healthy adults

For comparison with chronic pain patients, we analyzed blood data using the same DNN model for 252 patients who visited the Dementia Prevention Outpatient Clinic of the same age group without chronic pain. The estimated MMSE score in the control group was 27.2 points (i.e., it exceeded the cut off value for MCI), which was significantly lower in chronic pain patients (*p* = 0.0241). There were no significant differences in age or gender between the two groups (Table 6). This implies a potential risk of MCI in chronic pain patients.

**Table 6:** Comparison with control group.

		Pain patients		Normal adults		*p*	
		*N* (%)	Median (IQR)	*N* (%)	Median (IQR)		
Age, year		114	57.0 (49.8–67.0)	252	55.0 (50.0–61.0)	0.1848	
	<50	28 (24.6)		52 (20.6)			
	50-	41 (36.0)		121 (48.0)			
	60-	24 (21.1)		56 (22.2)			
	70-	15 (13.2)		20 (7.9)			
	80-	6 (5.3)		3 (3.8)			
Gender	Male	48 (42.1)		122 (48.4)		0.3085	
	Female	66 (57.9)		130 (51.6)			
Estimated MMSE, *p*			26.8 (25.6–27.8)		27.2 (26.1–28.0)	0.0241	*

## 4 Discussion

In this study, we used DNN to estimate the MMSE scores of chronic pain patients and found that more than half had a likelihood of MCI. Additionally, it revealed the characteristics of these patients.

### 4.1 Impact on cognitive function in chronic pain patients

Epidemiological analysis targeting community residents and pain clinic patients has reported that at least 50% of individuals living with pain experience cognitive impairments (4). In this study, when the DNN model was applied to patients visiting a pain clinic, an estimated 54% of them were identified as having MCI. Notably, elevated levels of neutral lipids were a significant characteristic of patients estimated to have MCI. Chronic pain is known to be related to both the cause and consequence of physical inactivity (26), with the prevalence of inactivity and lower back pain increasing among sedentary office workers (27). This relationship between pain and physical activity is further supported by the observation that alleviation and prevention of chronic pain can be achieved by transitioning from a sedentary lifestyle to an active one (28). In this study, while the neutral lipid levels in MCI patients did not exceed the standard values, they likely reflected reduced physical activity. Furthermore, irregular dietary habits and disrupted nutritional balance could also be contributing factors (29). Although data on this were not collected in this study, previous reports have indicated that individuals with higher body mass index (BMI) are at a higher risk of dementia (30), suggesting a connection between reduced activity and dementia risk. Our results suggest that a decrease in physical activity due to pain and dietary irregularities could lead to disturbances in lipid metabolism and pose a risk to cognitive function.

Surprisingly, the hemoglobin and hematocrit levels were significantly higher in the MCI group. While both groups fell within the standard range, these results differ from past reports showing a positive correlation between hemoglobin and MMSE scores (11). Dehydration may be a contributing factor in this finding. It’s worth noting that the patient population with chronic pain included a significant proportion of individuals in their 70s and 80s (13.2 and 5.3%, respectively). Although this study focuses on acute pain research, dehydration has been reported to increase pain sensitivity (31). A state of anxiety due to dehydration significantly amplifies pain-related brain activity, making individuals more sensitive to painful stimuli (32). It remains a future task to clarify whether mild dehydration can lower pain thresholds and investigate the fundamental mechanisms.

Moreover, the significantly elevated values of kidney and liver function parameters in patients estimated to have MCI indicate the need to consider the effects of polypharmacy, long-term medication use, and high-dose intake of medication for pain management. For instance, acetaminophen, commonly used either alone or in combination with other analgesics, may lead to liver function impairment with long-term or high-dose use (33). Similarly, non-steroidal anti-inflammatory drugs (NSAIDs), frequently employed in pain management, could potentially result in kidney function impairment (34). The elevated liver and kidney function values in patients estimated to have MCI may be attributable to drug-induced reactions. Furthermore, recent reports suggest that long-term opioid use is associated with an increased risk of dementia in chronic pain patients (35). As this study did not investigate the status of medication therapy, it is an area to be explored in future research.

### 4.2 Verification of the relationship between the questionnaire results and cognitive function

This study found a negative correlation between the BPI scores and estimated MMSE values, indicating that individuals with more severe pain complaints tend to have lower MMSE scores. BPI is a widely used clinical assessment tool (36). It reflects the concept of fear-avoidance beliefs, suggesting that negative emotions such as anxiety and depression induced by pain can not only significantly decrease activities of daily living (ADL) and quality of life (QOL) but also serve as triggers for mental disorders or mood disturbances. Such mental states can exacerbate the experience of pain, creating a vicious cycle (37).

Moreover, negative correlations were observed between PDAS and Locomo scores and estimated MMSE values. Individuals who experience significant impacts from daily life pain or have reduced mobility due to physical impairments tend to exhibit lower estimated MMSE scores. While the number of chronic pain sites is related to an increased risk of dementia, lifestyle and other confounding factors do not sufficiently explain this relationship (38). Although this study did not investigate lifestyle factors, it is essential to pay attention to the association with individuals who are affected in their daily lives by pain. Additionally, those who experience challenges in physical activities and mobility may be more prone to developing systemic metabolic disorders, as discussed in the previous section. Moreover, even when blood data results are within the normal range, chronic pain may pose a risk for MCI. This aligns with reports suggesting that lifestyle-related diseases can lead to vascular cognitive impairment due to atherothrombotic vascular disease (39). However, intriguingly, PDAS scores were significantly lower in the MCI group. While this might seem contradictory to the previous findings, age may be a factor to consider. As shown in Table 2, the MCI group was significantly older than the non-MCI group. Therefore, the MCI group may have answered in a way that did not reflect the impact of pain on daily life, given their initially lower activity compared to the non-MCI group. Conversely, activities such as “running” and “carrying heavy objects” may have a more substantial impact on highly active younger individuals.

### 4.3 Limitations of this study

Firstly, this study was conducted with a limited amount of data, and due to its cross-sectional design, it cannot establish causation. It does not account for the impact of other factors, such as medication therapy or detailed lifestyle, and is in need of more extensive sample sizes, longer-term follow-up investigations, and a broader assessment of various factors in future research.

Secondly, the study included all chronic pain patients who visited a pain clinic without considering the degree, site, or duration of pain. Subgroup analysis by pain severity, physical activity, sleep conditions, and other factors will be a future task.

## 5 Conclusion

More than half of chronic pain patients had a possibility of MCI. This may be related to chronic pain affecting daily life activities or to systemic metabolic disturbances caused by pain medications.

## Data availability statement

The raw data supporting the conclusions of this article will be made available by the authors, without undue reservation.

## Ethics statement

The studies involving humans were approved by the Ethics Committee for Research in the Life Sciences and Medicine involving individuals at Yokohama City University. The studies were conducted in accordance with the local legislation and institutional requirements. Written informed consent for participation was not required from the participants or the participants’ legal guardians/next of kin because this research did not involve any invasiveness or intervention, and only used existing information held by our own institution. Based on the ethical guidelines for life science and medical research involving human subjects, information about the research was disclosed to research subjects, and research subjects were guaranteed the opportunity to object to the research being conducted.

## Author contributions

MM: Conceptualization, Data curation, Formal analysis, Funding acquisition, Investigation, Methodology, Project administration, Resources, Writing – original draft, Writing – review & editing. LH: Formal analysis, Writing – review & editing. KS: Conceptualization, Methodology, Supervision, Writing – review & editing. MK: Conceptualization, Funding acquisition, Methodology, Supervision, Writing – review & editing.
